# The Esthetic Perception of Morphological Severity in Scaphocephalic Patients is Correlated With Specific Head Geometrical Features

**DOI:** 10.1177/10556656221111307

**Published:** 2022-07-03

**Authors:** Alessandro Borghi, P. Heutinck, N. Rodriguez-Florez, M. Koudstaal, F. Ruggiero, S. Ajami, S. Schievano, N.U.O. Jeelani, D. Dunaway

**Affiliations:** 1 UCL Great Ormond Street Institute of Child Health, London, UK; 2 4956 Great Ormond Street Hospital for Children, London, UK; 3 Erasmus University Hospital, Rotterdam, the Netherlands; 4 Universidad de Navarra, 54393 TECNUN Escuela de Ingenieros, Donostia-San Sebastián, Spain; 5 Ikerbasque, Basque Foundation for Science, Bilbao, Spain; 6 DIBIDEM, 9296 Alma Mater Studiorum University of Bologna, Bologna, Italy

**Keywords:** craniofacial morphology, cephalometry, computerized tomography

## Abstract

**Objective:**

To investigate the relationship between perception of craniofacial deformity, geometric head features, and 3D head shape analyzed by statistical shape modeling (SSM).

**Patients:**

A total of 18 unoperated patients with scaphocephaly (age  =  5.2  ±  1.1m)—6 were followed-up after spring-assisted cranioplasty (SAC) (age  =  9.6  ±  1.5m)—and 6 controls (age  =  6.7  ±  2.5m).

**Main Outcome Measures:**

3D head shapes were retrieved from 3D scans or computed tomography (CTs). Various geometrical features were measured: anterior and posterior prominence, take-off angle, average anterior and posterior lateral and horizontal curvatures, cranial index (CI) (cranial width over length), and turricephaly index (TI) (cranial height over length). SSM and principal component analysis (PCA) described shape variability. All models were 3D printed; the perception of deformity was blindly scored by 9 surgeons and 1 radiologist in terms of frontal bossing (FB), occipital bulleting (OB), biparietal narrowing (BN), low posterior vertex (LPV), and overall head shape (OHS).

**Results:**

A moderate correlation was found between FB and anterior prominence (r  =  0.56, *P* < .01) and take-off angle (r  =  − 0.57, *P *< .01). OB correlated with average posterior lateral curvature (r  =  0.43, *P* < 0.01) similarly to BPN (r  =  0.55, *P* < .01) and LPV (r  =  0.43, *P* < .01). OHS showed strong correlation with CI (r  =   − 0.68, *P* < .01) and TI (r  =  0.63, *P*< .01). SSM Mode 1 correlated with OHS (r  =  0.66, *p* < .01) while Mode 3 correlated with FB (r  =   − 0.58, *P* < .01).

**Conclusions:**

Esthetic cranial appearance in craniofacial patients is correlated to specific geometric parameters and could be estimated using automated methods such as SSM.

## Introduction

Sagittal craniosynostosis affects 1 born every 1700^1^ and is the most common presentation of non-syndromic craniosynostosis. Because of compensatory overgrowth in the plane parallel to the sagittal suture, the skull acquires a typically elongated boat-like shape. The principal treatment goal is correction of head deformity, which is achieved by means of a range of surgical techniques (total calvarial remodeling,^2^ strip craniectomy,^3^ and springs^4^).

Spring-assisted cranioplasty (SAC) has proved to be a safe and reliable technique to perform minimally invasive cranial reshaping by means of implantable metallic distractors which exert force on the pediatric calvarium and promote remodeling.^4,5^ Recent studies have proved that numerical methods, such as statistical shape modeling (SSM)^6^ and finite element analysis^7,8^ can help predict the outcome of SAC. These methodologies describe the relationship between the SAC surgical outcome (in terms of geometrical shape the patient calvarium achieves post-surgery) with osteotomy size and locations as well as the choice of distraction force; however, the connection between localized change in shape and qualitative esthetic outcome has never been described, due to difficulties in consistently quantifying the latter.

Several studies in the literature have quantified the severity of deformity in patients affected by non-syndromic craniosynostosis. Panchal et al. compared esthetic outcomes after extended strip craniectomy or total calvarial remodeling with normal subjects by means of photographic assessment.^9^ Kluba et al. analyzed and compared the parental and professional perception of outcome in children treated for isolated as well as complex craniosynostosis, finding that complex craniosynostosis yields a more pronounced discrepancy in outcome assessment between parents and clinicians.^10^ A similar scoring was used by Fearon et al. to explore connection between photographic assessment of scaphocephaly severity and cephalic index in sagittal craniosynostosis patients^11^: their results showed that the posterior skull height correction had an effect on scoring, which was not depicted by cranial index (CI) alone. A recent work from Oxford^12^ aimed at reliably scoring the surgical correction of sagittal craniosynostosis using a visual analogue scale grading skull shapes in patients having total or subtotal calvarial remodeling according to 5 different aspects (narrow elongated skull, frontal bossing [FB], temporal pinching, occipital bullet [OB], and overall head shape [OHS]). The same approach was used by Salokorpi et al. for assessing the long-term esthetic outcomes of patients treated for scaphocephaly^13^: images of patients who reached adulthood as well as age-matched controls were presented to clinical personnel as well as laypersons, who visually graded the attractiveness while blind to the nature of the subjects.

Studies on aesthetic surgical outcomes mostly rely on the use of 2D clinical pictures. Such imaging modality, although providing standardized subject views, does not allow the raters to assess the marked changes in 3D shape experienced by patients treated for craniofacial malformations - such as babies affected by scaphocephaly. Such 3D changes can be measured by standard craniometrics measurements^14^ as well as using more complex shape modeling techniques.^15^

A connection between geometric head features and esthetic perception of severity of scaphocephaly has never been investigated: we present here data on esthetic assessment on 3D-printed calvarial models and examine a correlation with several geometric measurements performed on 3D.

## Materials and Methods

### Patient Population

A total of 24 patients were recruited for this study. 3D head scans (acquired on table using a Rodin4D surface scanner—Pessac, Aquitaine, France) were acquired with a handheld scanner for patients affected by scaphocephaly before the SAC procedure (n  =  18, PRESAC); for a subset of these patients (n  =  6, POSTSAC), long-term post-op scans were also retrieved. Computed tomography (CT) scans relative to patients who presented at Great Ormond Street Hospital (GOSH) for non-craniofacial procedures were retrieved (n  =  6, NORM): 3D head shapes were extracted using Mimics (Materialise).

**Figure 1.**
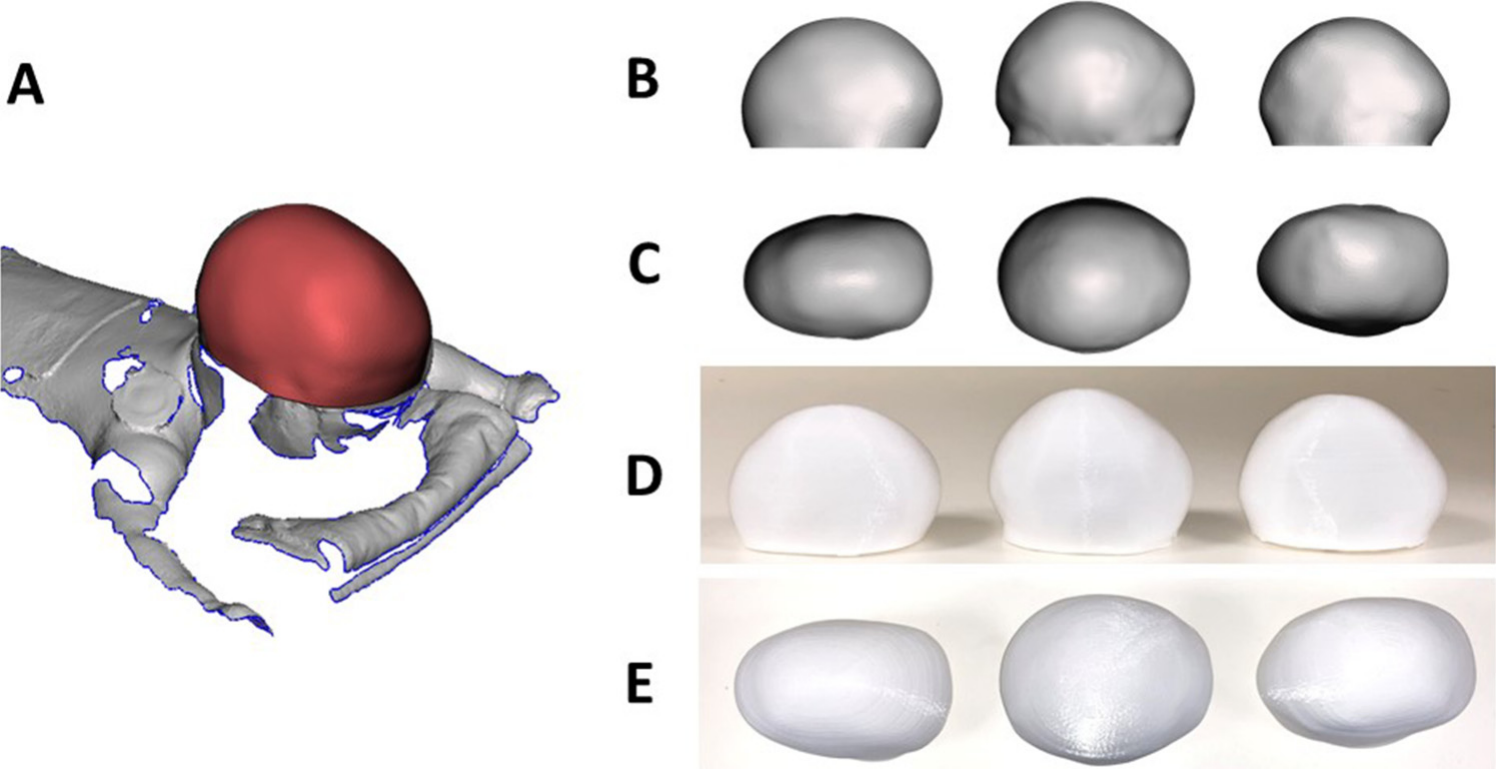
(A) Sample of 3D scan of a patient retrieved while on table, with the region of interest in red; sample of 3D models used to assess the perception of severity of scaphocephaly: 3D virtual model in side (B) and top view (C) with relative 3D-printed models in side (D) and top view (E).

For each head shape model, stereolithography (STL) files were exported as 3D surface meshes and processed using Meshmixer (Autodesk Research). Each model was smoothed and cut using a plane passing through the nasion and both tragion points (Figure 1A). A more detailed description of the methodology is reported in Tenhagen et al.^15^

### Perception Study

All 30 head models were rescaled (Figure 1B and C) to ensure the occipitofrontal diameter of each head (maximum anterior-posterior dimension) was 10cm, in order to maintain head ratios but unify size among the population. Each head model was assigned a random number from 1 to 30 (which was added at the bottom of the model); each head was 3D printed in polylactic acid (PLA—Figure 1D and E) using a Makerbot Replicator 3D Printer (Makerbot).

A questionnaire was produced and presented to 10 assessors (9 surgeons—4 plastic surgeons, 3 neurosurgeons, 2 maxillofacial surgeons—1 neuroradiologist) who assessed each head model according to 5 aspects (outcomes): FB, OB, biparietal narrowing (BPN), low posterior vertex (LPV), and OHS. Table 1 reports a list of abbreviations. Each assessor was presented with the models, allowed to assess a few of them randomly to familiarize with the models and informed that each 3D-printed model was relative to a GOSH patient.

**Table 1.** List of Abbreviations Used Throughout the Text.

Abbreviations	Definitions
CI	Cranial Index
TI	Turricephaly Index
FB	Frontal Bossing
OB	Occipital Bulleting
BPN	Biparietal Narrowing
LPV	Low Posterior Vertex
OHS	Overall Head Shape

Each outcome was recorded on a scale from 1 to 100 as a measure of the severity of the deformity, with 1 corresponding to a normal head shape while 100 corresponding to a very severe deformity. Each score was recorded and average scores for each model and each outcome were calculated.

### Analysis of Geometrical Features

**Figure 2.**
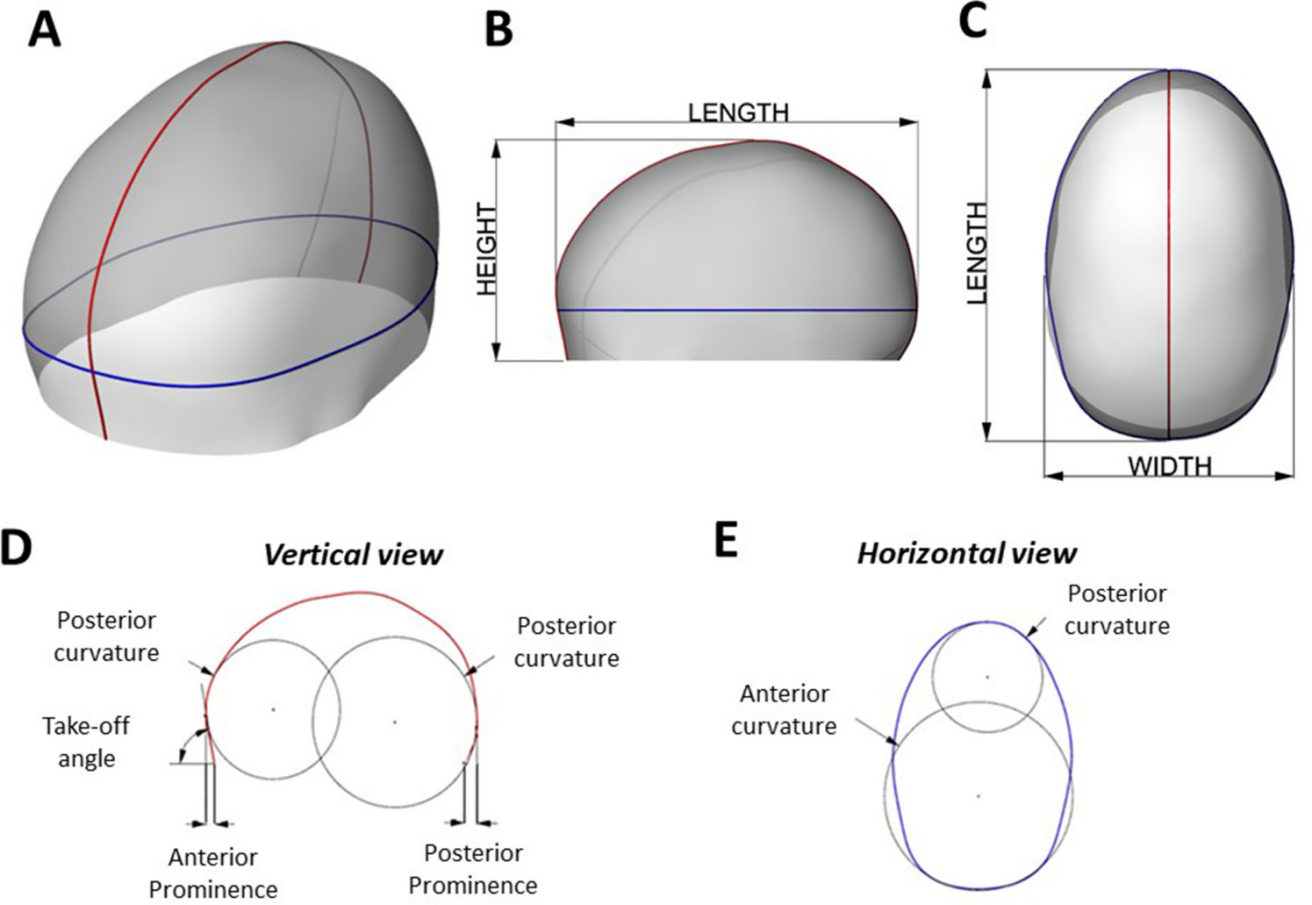
(A) Sample of 3D head virtual model visualizing the vertical section (in red) and horizontal section (in blue) used for the geometrical analysis; (B) side view and (C) top view of the 3D head sample – (D) side view of the vertical section, showing the measurement of anterior prominence, posterior prominence, take-off angle, frontal vertical curvature, and posterior vertical curvature. (E) Top view of the horizontal section showing frontal horizontal curvature and posterior horizontal curvature.

Each model STL file (Figure 2A) was analyzed to extract geometrical information. Each shape was cut along the midline (to perform measurements on the lateral projection “silhouette”—red on Figure 2B and C) and on a horizontal plane passing through the most anterior point of the forehead (to perform measurements on the horizontal projection—blue on Figure 2B and 2C).

From the lateral projection (Figure 2D), the following geometrical parameters were quantified:

- Anterior prominence: Horizontal distance between the nasion and the most prominent point of the forehead.
- Posterior prominence: Horizontal distance between the model’s most posterior base point and the most prominent point of the occiput.
- Take-off angle: Angle defined by the most prominent point of the forehead, the nasion, and the horizontal plane
- Average frontal vertical curvature: The average curvature of the anterior half silhouette.
- Average posterior vertical curvature: The average curvature of the posterior half silhouette.

From the horizontal projection (Figure 2E), the following geometrical parameters were quantified:

- Average frontal horizontal curvature: The average Gaussian curvature of the anterior half projection.
- Average posterior horizontal curvature: The average Gaussian curvature of the posterior half projection.

For each model, height, length, and width were measured (Figure 1B and 1C). CI was calculated as width over length; turricephaly index (TI)^16^ was calculated as height over length.

### Statistical Shape Modeling

DEFORMETRICA (www.deformetrica.org - a framework for statistical analysis of complex shapes extracted from 3D anatomical images) was used to assess shape variation in this cohort: a template (population average head shape) was calculated and variations of each patient sample from the template were extracted.^32^ Principal component analysis (PCA), which captures the shape variance around the template by extracting the deformations required to turn the template shape back to each individual shape (shape vectors—Pennec, 2009), was performed. Each subject can thus be described by the mean shape and specific shape deformation vectors (variation modes).

### Statistical Analysis

Univariate correlation between outcomes and geometrical features and outcomes and SSM shape modes was analyzed by means of Pearson’s correlation. For each of the analyses, the significance level was set at α  =  0.05.

## Results

Scans relative to 24 GOSH patients were acquired: 18 scaphocephaly patients undergoing SAC were scanned at an age of 5.2  ±  1.2 months (range: 3.3-7.4); a subset of these patients (n  =  6) were scanned again postoperatively after spring removal at an age of 6.7  ±  2.5 months (range: 3.1-12.2); 6 patients who received head CT scans with no craniofacial indication were also included (age  =  6.7  ±  2.5 months, range: 3.1-12.1). A total of 30 head shapes were included in the study.

Outcome ratings were gathered: FB, OB, BPN, LPV, and OHS were averaged throughout all the ratings for each patient.

**Figure 3.**
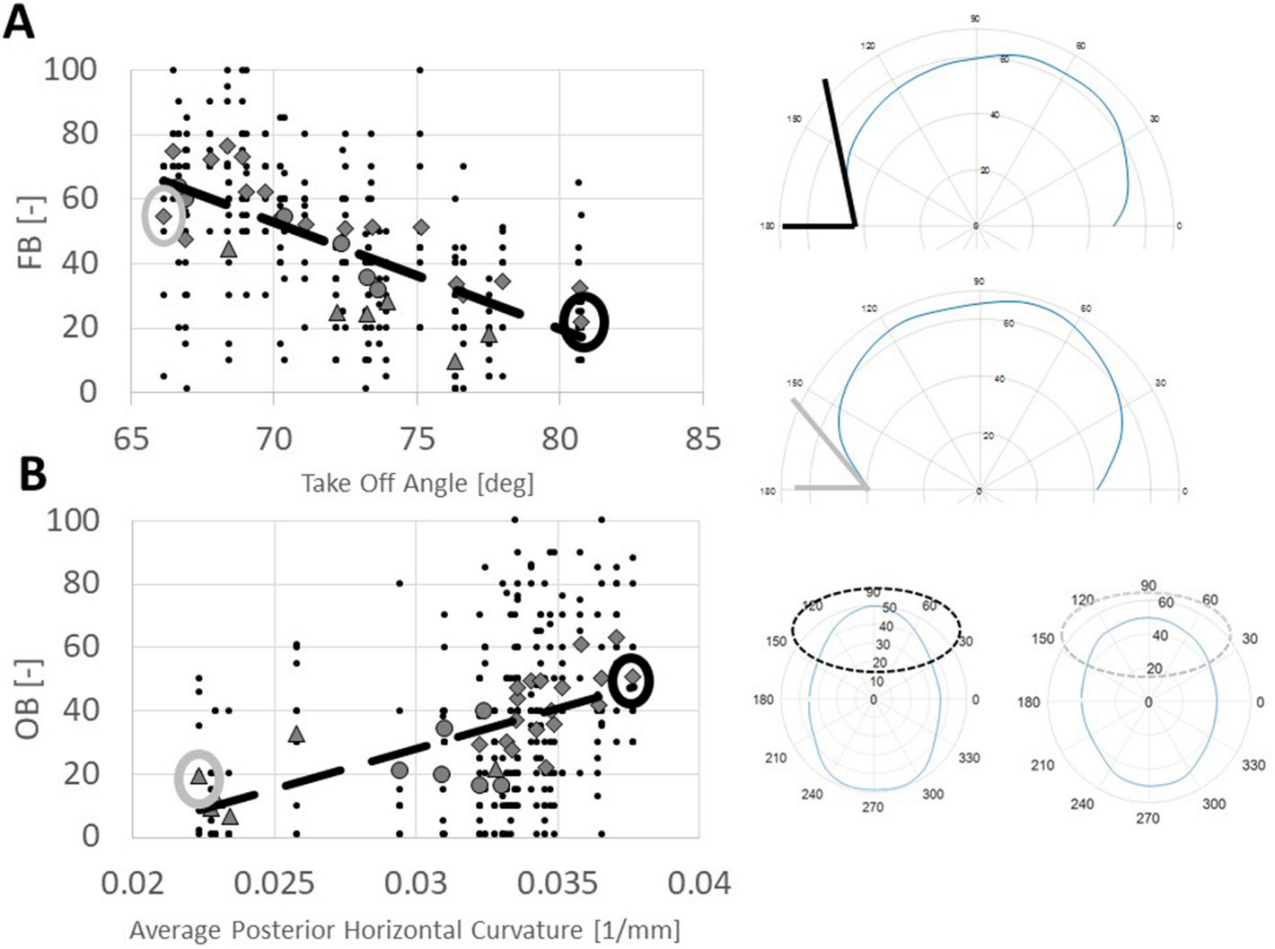
(A) Correlation between take-off angle and frontal bossing (FB). Black dots are all the individual scores while the dotted line shows linear correlation; different markers denote the average score for each subject, subdivided into the 3 subgroups (diamond  =  PRESAC, triangle  =  NORM, and circle  =  POSTSAC); on the right, example of the patients having highest (top) and lowest (bottom) TOFF. (B) Correlation between average posterior horizontal curvature and occipital bulleting (OB). Black dots are all the individual scores while the dotted line shows linear correlation; different markers denote the average score for each subject, subdivided into the 3 subgroups (diamond  =  PRESAC, triangle  =  NORM, and circle  =  POSTSAC); on the right, example of the patients having highest (top) and lowest (bottom) average posterior horizontal curvature.

Univariate correlations between the outcomes described earlier and the geometrical features (anterior prominence, posterior prominence, take-off angle, average frontal vertical curvature, average posterior horizontal curvature, average frontal vertical curvature, and average posterior vertical curvature) were performed. Table 2 shows, for each outcome, univariate correlations with the geometrical features. FB is moderately correlated with the take-off angle (r  =   − 0.57, *P* < .001, Figure 3A) while the average posterior horizontal curvature shows moderate correlation with OB (r  =  0.43, *P* < .001, Figure 3B), LPV (r  =  0.43, *P* < .001) and BPN (r  =  0.55, *P* < .001), and strong correlation with OHS (r  =  0.67, *P* < .001).

**Table 2.** Univariate Correlation Between the Outcomes and the Geometrical Factors, the Craniometric Indices, and Statisitcal Shape Modeling (SSM) Modes 1 to 3.

r_coeff_	Anterior Prominence	Take-Off Angle	Posterior Prominence	Average frontal horizontal curvature	Average Posterior Horizontal curvature	Average frontal vertical curvature	Average posterior vertical curvature	CI	TI	Mode 1	Mode 2	Mode 3
FB	0.56*	−0.57*	−0.17*	0.14*	0.40*	0.39*	0.19*	−0.37*	0.30*	−0.28*	−0.15*	−0.58*
OB	0.06	−0.06	0.13*	0.31*	0.43*	0.11	0.24*	−0.43*	0.40*	−0.43*	0.11*	−0.19*
BPN	0.01	−0.05	0.05	0.45*	0.55*	0.18*	0.42*	−0.61*	0.49*	−0.54*	−0.05	−0.27*
LPV	0.05	−0.05	−0.03	0.27*	0.43*	0.13*	0.26*	−0.44*	0.43*	−0.44*	−0.02	−0.22*
OHS	0.04	−0.05	0.00	0.48*	0.67*	0.21*	0.44*	−0.68*	0.63*	−0.66*	−0.01	−0.29*

* Significant correlation (*P* < .05).

Abbreviations: BPN, biparietal narrowing; CI, cranial index; FB, frontal bossing; LPV, low posterior vertex; OHS, overall head shape; TI, turricephaly index.

Correlation with craniometric indices shows a negative correlation for all the indices when compared with CI and a positive correlation when compared with TI (Table 2). Strongest correlation is between OHS and CI (r  =   − 0.67, *P* < .001) and OHS and TI (r  =  0.63, *P* < .001).

**Figure 4.**
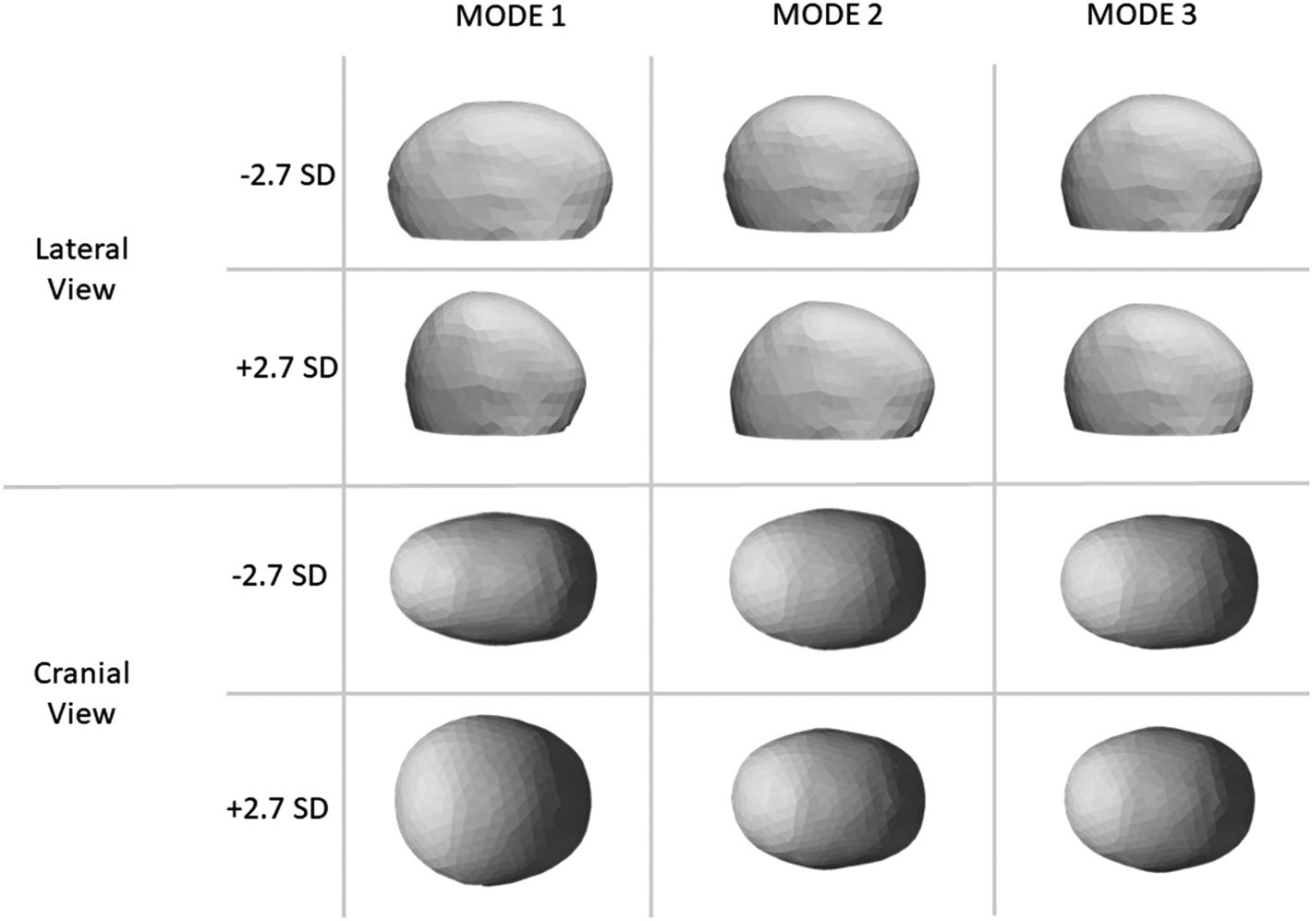
Visualization of the first 3 modes of variation after applying principal component analysis (PCA) to the patient population analyzed, lateral view (first and second rows), and cranial view (third and fourth rows). Each mode of variation is represented by shape models varying between − 2.7 standard deviation (SD) and 2.7 SD of the respective shape mode.

**Figure 5.**
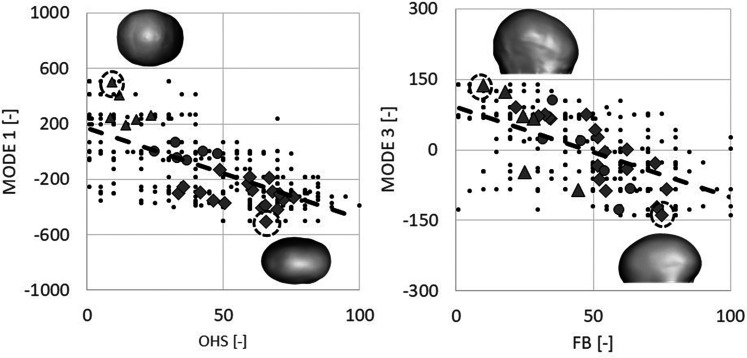
On the left: Correlation between Statistical Shape Modeling (SSM) Mode 1 and overall head shape (OHS). Black dots are all the individual scores while the dotted line shows the linear correlation; different markers denote the average score for each subject, subdivided into the 3 subgroups (diamond  =  PRESAC, triangle  =  NORM, and circle  =  POSTSAC; the subject with higher and lower Mode 1 value are shown as insets). On the right: correlation between SSM Mode 3 and frontal bossing (FB). Black dots are all the individual scores while the dotted line shows the linear correlation; different markers denote the average score for each subject, subdivided into the 3 subgroups (diamond  =  PRESAC, triangle  =  NORM, and circle  =  POSTSAC; the subject with higher and lower Mode 3 value are shown as insets, facing right).

SSM and PCA were used to extract principal modes of variation throughout the population. Figure 4 shows the shape variations of the populations analyzed for Modes 1, 2, and 3. A moderate correlation was found between PCA Mode 1 and BPN (r  =   − 0.54, *P* < .001) while a strong correlation was found with OHS (r  =   − 0.66, *P*< .001, Figure 5—left) as reported in Table 2. A moderate correlation was found between PCA Mode 3 and FB (r  =   − 0.58, *P* < .001, Figure 5—right) as reported in Table 2.

## Discussion

The assessment of surgical outcomes of craniofacial surgery is challenging due to the esthetic component of the treatment, which proves difficult to quantify. This is particularly true in the case of correction of sagittal craniosynostosis, where compensatory overgrowth of the patent sutures causes the skull to gain a progressive boat-shaped deformity in the majority of the cases, which is characterized by specific features, such as FB, OB, BPN, and presence of a LPV.^12^ The goal of this study was to draw a correlation between geometrical calvarial features and perception of scaphocephaly severity in a mixed patient population. Computer modeling can be used to predict scaphocephaly correction outcomes;^6,7,17^ however, since the final aim is to achieve esthetic improvement, a relationship between the geometrical output of computer models and predicted esthetic outcome is necessary to adopt these numerical models successfully in clinical practice. SSM has shown to be a valuable tool for the assessment of head shape: it was able to predict the OHS rating trend in this patient population and could be therefore used as a tool for automated assessment of surgical planning predictions.

Efforts in the past have been made to attempt both qualitative and quantitative evaluation of scaphocephaly correction: Taylor et al.^18^ performed a review of 238 patients treated for unicoronal craniosynostosis and used the Whitaker classification method to classify in terms of esthetic outcomes. Such classification method stratifies patients according to the type of secondary operation required to achieve esthetic normality. The authors correlated clinical variables such as age of intervention, choice of bone grafting and presence of preoperative compensatory bossing, nasal deviation and midface retrusion with onset of supraorbital retrusion, temporal hollowing, and high Whitaker classification at the long-term follow-up. Bendon et al.^12^ used similar assessment criteria to this work to assess the outcome of surgery of 42 consecutive cases of sagittal synostosis using a visual scale. Clinical assessors deemed that the type of calvarial remodeling was an indicator of outcome for all aspects of skull shape. Fearon et al.^11^ used a combined quantitative-qualitative approach to correlate cephalic indexes with perceived severity score in a scaphocephaly population by means of standardized preoperative photographs. Other studies have focused on qualitative assessment of the surgical outcome perception from the point of view of the parents^10,19^ or the patients themselves once adults.^13,20^ Quantitative assessment has involved the use of craniometric measurements retrieved by means of cephalometric landmarks^14,21^ which allow quantification of the calvarial shape change. Recent works have introduced assessment of shape change and outcome of surgical craniosynostosis correction by means of SSM.^6,15,22–24^

Surgical planning of scaphocephaly correction has been performed in the past by means of 3D-printed models^25^ as well as using virtual planning ^26^; more recently, finite element modeling (FEM) was introduced to plan cranial reshaping^27–29^ as well as SAC.^7,17,30^ FEM allows virtual planning of surgical correction strategies by allowing to test the effect of osteotomy locations and distractor selection on the overall calvarial shape as well as localized features. However, cranial augmentation alone may not be sufficient to ensure a “good aesthetic outcome”.

This work entangles the relationship between a wide range of geometrical calvarial features and expert assessment of head shape in a mixed population of subjects. Correlation with geometrical features showed that severity of FB is highly correlated to frontal take-off angle, as reported by Seruya et al.^14^ while average posterior horizontal curvature is correlated to OB, BPN, and LPV: therefore, more pronounced scaphocephaly is perceived because of a high curvature of the back of the head. Although the connection between vertex position and horizontal curvature may not be obvious, it is likely that severely scaphocephalic head have several prominent features. Correlation with craniometric indices showed that low CI and high TI are correlated with severe values of all the outcomes; therefore, these indices describe well severity of scaphocephaly. Only another work in the literature relates CI index to observer scoring^11^ and no correlation was found. However, only 3 groups were observed and absolute z-score was analyzed, hence patients with very high and very low CI are grouped together, decreasing the chance of having statistical correlation.

The results of this study suggest that to improve head appearance, surgical correction of scaphocephaly should aim at increasing the take-off angle to improve the perception of FB and augment posterior head size, thus decreasing posterior curvature, in order to improve the perception of OB and BPN as well as of OHS. CI and TI should be monitored pre- and postoperatively as they are both strong predictors of OHS perception.

A further interesting outcome of this study is that head shape perception can be predicted by means of SSM. Such methods has been used in the past to describe anatomical variations^23,31,32^ in a patient population, as well as to assess and correlate shape with surgical outcomes.^15,22–24,33^ The shape variations shown in Figure 4 highlight how the main mode of variations of this population qualitatively relate to the severity of scaphocephaly (Mode 1) and forehead prominence (Mode 3). The OHS rating correlated with the Mode 1 of variation within the patient population (r  =   − 0.66) and such correlation was comparable with that of the CI (r  =   − 0.68), and TI (r  =  0.63) as well as average posterior horizontal curvature (r  =   − 0.67). This suggests that SSM, which captures overall 3D shape variations as opposed to simplified linear dimensions or ratios, provides an accurate representation of the complexity of 3D head shapes (as recently reported by Heuntick et al.^23^) and has the potential of predicting clinical perception comparably to standardized craniometric parameters (such as TI and CI): the current model was created using 30 shapes and it is likely that a model based on a larger population would further improve the correlation between OHS and PCA modes.

Main limitation of this study lies in the limited No. of assessors and limited No. of patients assessed. All of the assessors (10) were craniofacial specialists, either consultants or trainees. Each assessor was initially trained by viewing several random models for a few minutes before starting the full assessment. Although a small cohort of patients (24, for a total of 30 head models) was considered, statistical differences in scores were found between the different subgroups for all the outcomes. Furthermore, the results showed a significant correlation between the geometrical features and the perception outcomes.

This study shows that specific geometrical features are related to severity perception of scaphocephaly: the same protocol could be used to achieve a more quantitative understanding of the esthetic outcomes in other reconstructive interventions in craniofacial surgery.
